# Communication between the leaflets of asymmetric membranes revealed from coarse-grain molecular dynamics simulations

**DOI:** 10.1038/s41598-018-20227-1

**Published:** 2018-01-29

**Authors:** Jonathan Shearer, Syma Khalid

**Affiliations:** University of Southampton, Southampton, SO17 1BJ United Kingdom

## Abstract

We use coarse-grain molecular simulations to investigate the structural and dynamics differences between an asymmetric and a symmetrical membrane, both containing beta barrel transmembrane proteins. We find in where the dynamics of the two leaflets differ greatly, the slowest leaflet dominates the structural effects and importance of protein-lipid interactions.

## Introduction

Lipids in biological membranes are arranged bilayers, and contain proteins of a range of different sizes and architectures that span the full membrane or just one leaflet of the bilayer. Aside from their biological importance, these membrane proteins have provided the inspiration for devices used in bionanotechnology for applications such as DNA sequencing^1–4^. Hence understanding the molecular-level dynamics that occur within membranes that contain natural proteins is key from a number of perspectives^5,6^. In recent years it has become apparent that biological membranes are far more complex in their composition and patterns of behavior than suggested by traditional textbook models. In particular, these membranes are often very crowded with proteins and when these proteins diffuse laterally, they do so in complex with lipids^7–10^. The diffusion of proteins within membranes has been the focus of a number of recent experimental and computational studies, which have shown that the influence of proteins on lipid diffusion and membrane structure can extend up to ~6 nm^10–13^. While computational studies of protein-lipid interactions for symmetrical membranes are common^11–15^, similar work for asymmetric membranes is rare. Such studies are important for the understanding of bacterial membranes, which often have different lipid compositions in the two leaflets^16^. One bacterial asymmetric membrane of particular importance is the outer membrane of Gram-negative bacteria, which acts as a barrier to the chemical attack of the rest of the cell envelope^17–19^. Understanding such complex bacterial systems is key in the ongoing fight against bacterial resistance. Coarse-grained simulations have been shown to accurately reproduce physical properties of atomistic membrane models^20–23^, while being capable of accessing events over biologically relevant time- and length-scales, thus they provide an ideal methodology for studying protein and lipid diffusion and membrane structure^11,13,22,24^.

## Results

Here we investigated the structural and dynamics differences between the outer membrane and a symmetrical mixed phospholipid membrane, which contained a transmembrane protein; OmpA (small) or OmpF (large), additional details of the two proteins (Figure S1) are given in the methods section. Our model of the outer membrane was composed of an upper leaflet of lipopolysaccharide (LPS) molecules and a mixture of phospholipids (90% 16:0–18:1 phosphoethanolanime (POPE), 5% 16:0–18:1 phosphoglycerol (POPG) and 5% cardiolipin) in the lower leaflet. The mixed phospholipid membrane had the same composition as the lower leaflet of the outer membrane model. Here we investigated the structural and dynamics differences between the outer membrane and a symmetrical mixed phospholipid membrane with the same composition as the lower leaflet of the inner membrane. Given that the LPS layer has been reported to have a diffusion value an order of magnitude less than most phospholipid bilayers^16,25^ we expected this asymmetry in dynamics may lead to differences in the effect of a protein on its local environment. The slow diffusion of LPS is mainly a result of inter-lipid binding by Ca^2+^ ions. The order and density analysis for the mixed phospholipid system showed that there is a ~3 nm region around the protein in which the order of lipid tails and membrane thickness is reduced as a result of the disordered packing of the lipids bound to the rough protein surface (SI, Figures S2–S6). These observations compare well to previous atomistic simulations of nanopores in DOPC membranes^14^. For the symmetric membrane any significant changes in order parameters and lipid density between leaflets is confined to the local region around the protein.

**Figure 1:**
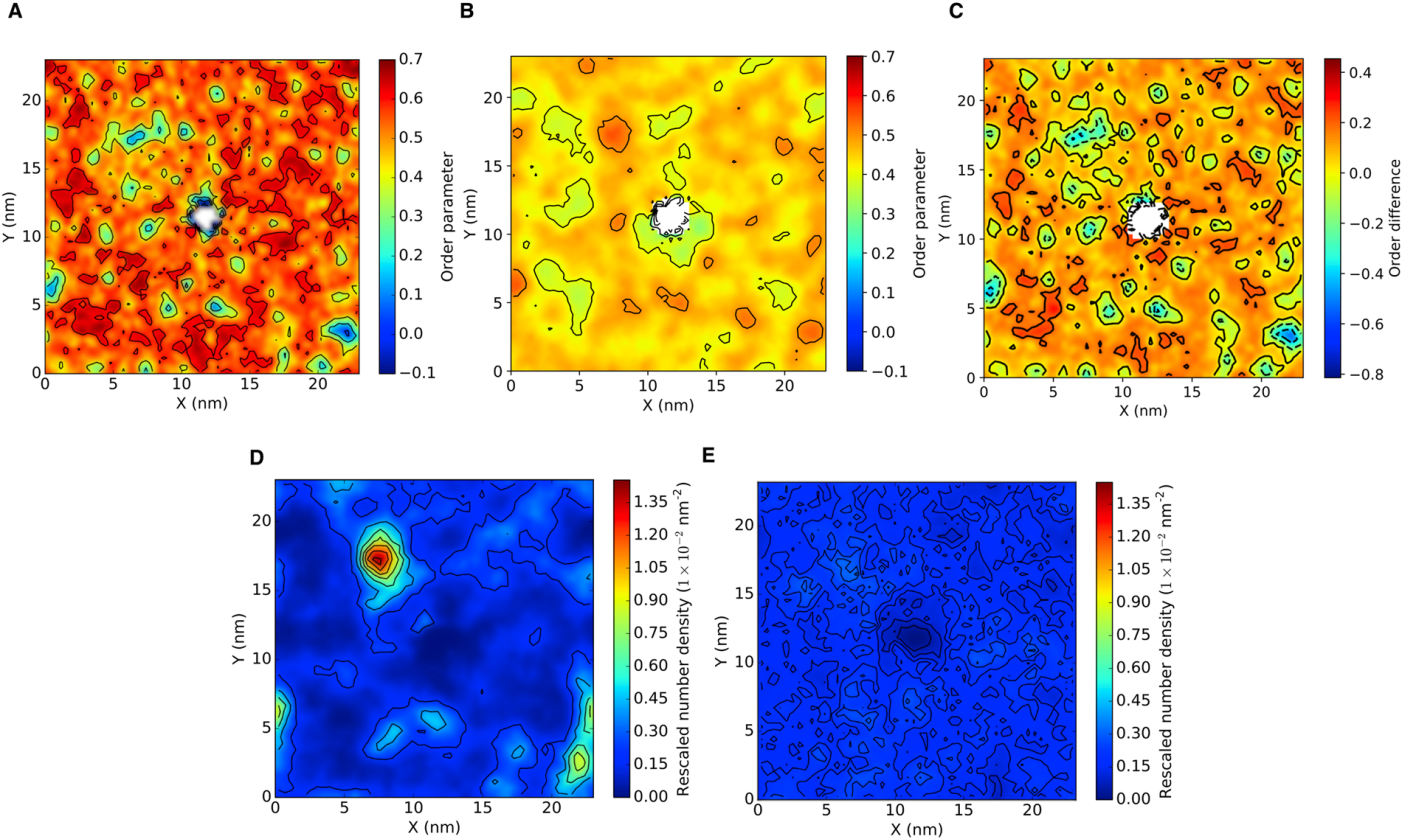
Order analysis of the lipid tails of (**A**) lipopolysaccharide, POPE and the (**C**) difference between lipopolysaccharide and POPE order parameters in the outer membrane. (**D**,**E**) Density maps of cardiolipin in the outer and mixed phospholipid membrane, respectively. The density was measured using one phosphate particle per lipid and all values normalized by the number of lipids in a given leaflet. Each system was centered around a transmembrane OmpA.

The order values of the LPS leaflet vary greatly across the membrane from magnitudes of around 0.15–0.70, which matches experimental order parameter measurements of multiple phases of LPS^26^. Comparison of the order analysis of the lipids in the upper and lower leaflets of the outer membrane showed that regions of high tail disorder in the LPS layer often corresponded to regions of high tail order in the same region of the lower leaflet (see Fig. 1A and B). Given the slower dynamics of the upper leaflet compared to the lower leaflet it is reasonable to deduce that the order of the tails in the upper leaflet determined the order of tails in the lower leaflet.

The density maps of each lipid type in the lower leaflet showed an increase in density below regions of high disorder in the upper leaflet. This observation was particularly apparent for the density map of cardiolipin, which clustered in regions corresponding to high disorder in LPS tails. A hypothesis for the observed nano-domains in LPS was that there were voids in the sugar head group packing due to the large size of LPS, combined with low conformational freedom stemming from Ca^2+^ inter-lipid binding. The lack of conformation freedom was highlighted by the density map in which the trajectory was centered around each protein (SI, Figure S7). Voids in LPS head group packing could mean that LPS tails splayed into the voids to maintain the bilayer surface, which lead to high LPS tail disorder and varying local leaflet thickness (Figure S8). Conversely, below regions of high LPS tail disorder the lipid tails extended into the upper leaflet to maximize the hydrophobic interaction with the disordered upper leaflet, which would explain the increased density and lipid tail order in the regions of high LPS disorder.

**Figure 2:**
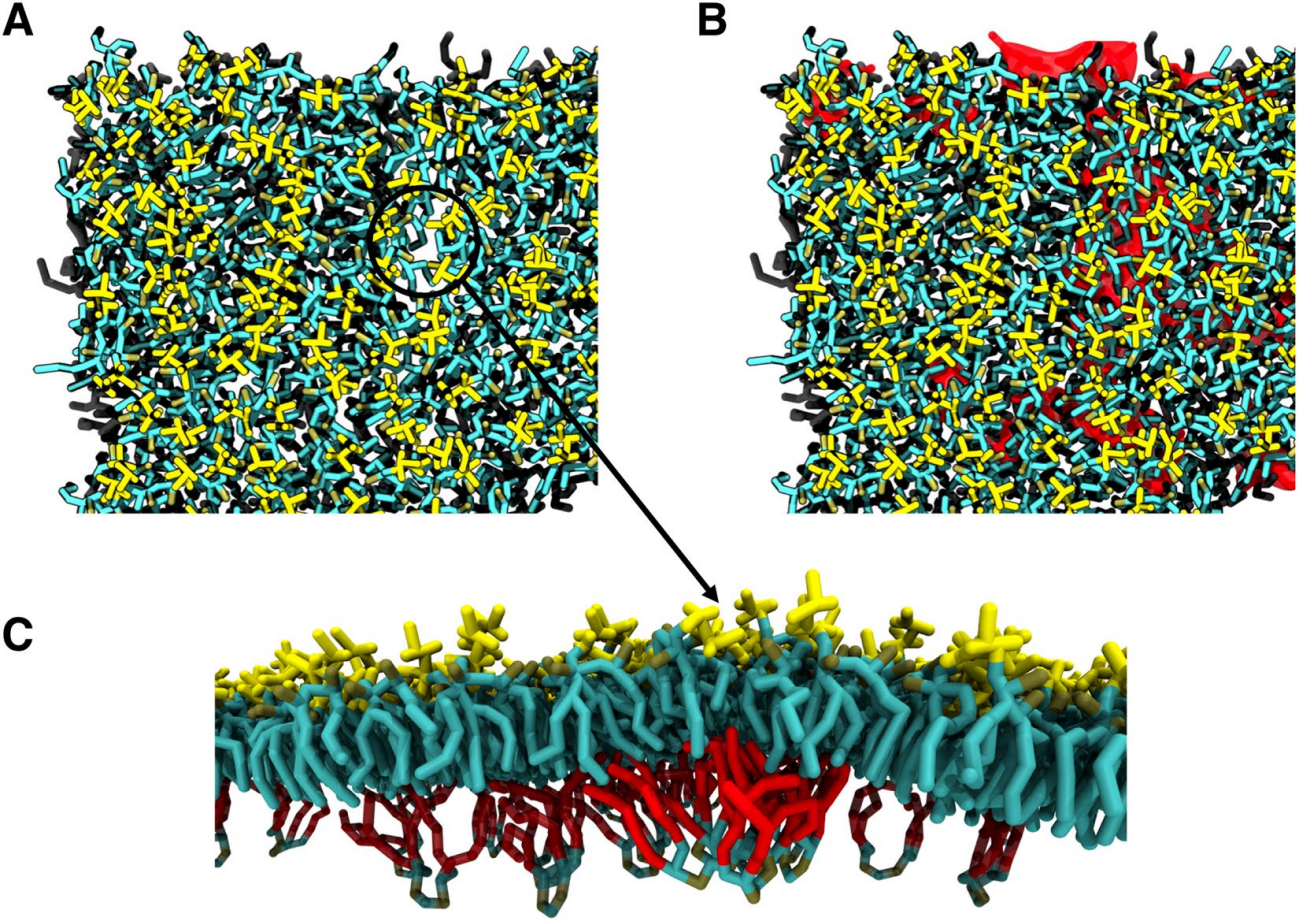
Top down view of the upper left quarter of the outer membrane with a transmembrane protein; OmpA (**A**) with and (**B**) without cardiolipin (highlighted in red) shown to emphasis the void in lipopolysaccharide sugar-sugar packing. (**C**) Side view of cardiolipin cluster (tails highlighted in red) below a void in LPS sugar-sugar packing. The system was centered on OmpA.

**Figure 3:**
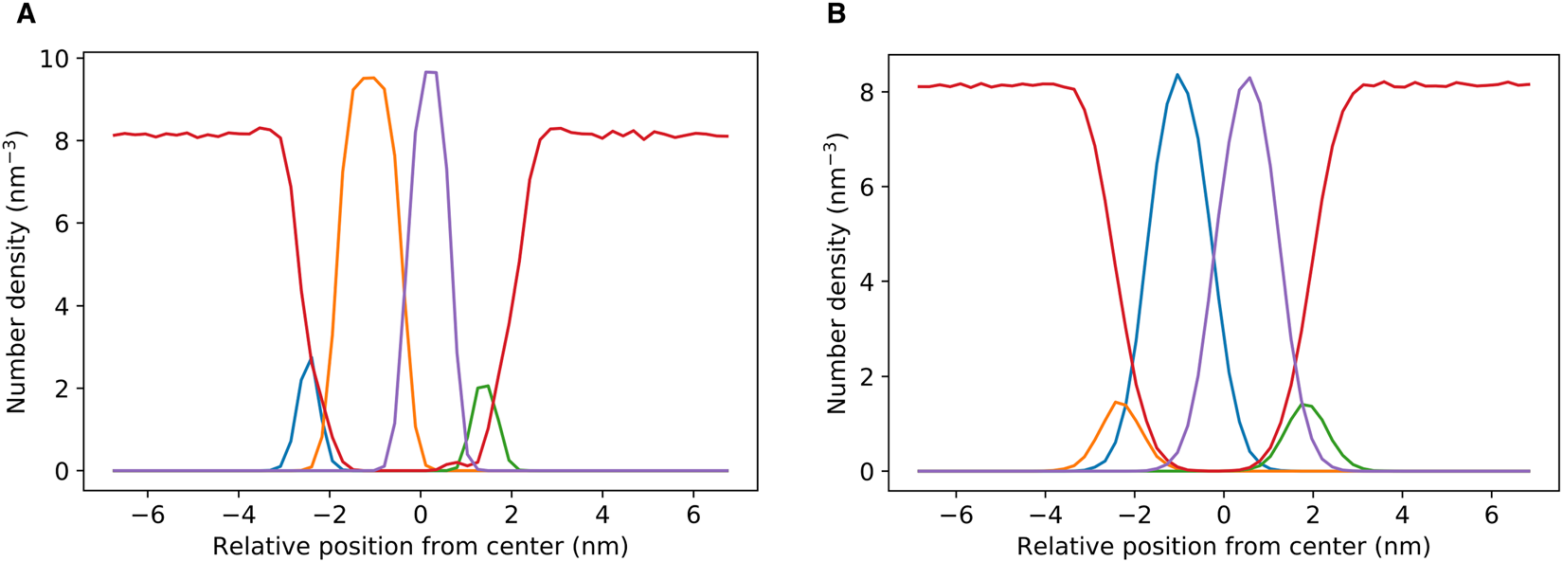
Partial density profile in the z direction for a 3 × 3 nm patch of the OmpA (**A**) outer membrane and the (**B**) mixed phospholipid systems, relative to the center of mass of each membrane - note that in (**A**) LPS was the upper leaflet of the membrane. The membrane patch had the bounds in the xy plane of 6 < x < 9 nm and 16 < y < 19 nm relative to a trajectory centered about the membrane OmpA (refer to Fig. 1). Key: red, water; blue, lower leaflet phosphate groups; yellow, lower leaflet tails; purple, upper leaflet tails; green, upper leaflet phosphate groups.

Visualization of the upper left quarter (Fig. 2) of the outer membrane (see Fig. 2A) for one of the frames in the simulation showed that there are a number large voids in the sugar packing of the LPS layer below regions where cardiolipin would be. Once the cardiolipin that was ignored in Fig. 2A was added (Fig. 2B) it was clear the voids in LPS packing were directly above the largest clusters of cardiolipin. Looking at the cross-section of the largest LPS packing mismatch, it can be seen that the cardiolipin tails appeared to be filling the void in the upper leaflet caused by the poor LPS packing. While clustering of PE and PG lipids is observed below regions of poor LPS packing, cardiolipin seems to preferentially pack into these voids. This can be reasoned using entropic arguments, as cardiolipin is the largest lipid in the lower leaflet and so the loss in entropy of cardiolipin ordering and clustering into the LPS voids is less than the clustering of PE or PG lipids. Similar trends were also observed for an outer membrane system with no embedded protein (SI, Figure S9). To analyze the degree of interdigitation between leaflets in the outer membrane we calculated the partial density in the region of the membrane circled in Figs 2 and 3. For the sake of comparison, the partial density profile was also determined for a similarly sized region of the mixed phospholipid system. For the symmetrical mixed phospholipid membrane, the density profile showed that the tails in each leaflet penetrate into their opposing leaflets by equal amounts (Fig. 3B). In the density profile of the outer membrane (Fig. 3A) the tails in the lower leaflet reached further into the upper leaflet than the LPS tails reached into the lower leaflet; suggesting that partial interdigitation occured between lipid tails in each leaflet.

**Figure 4:**
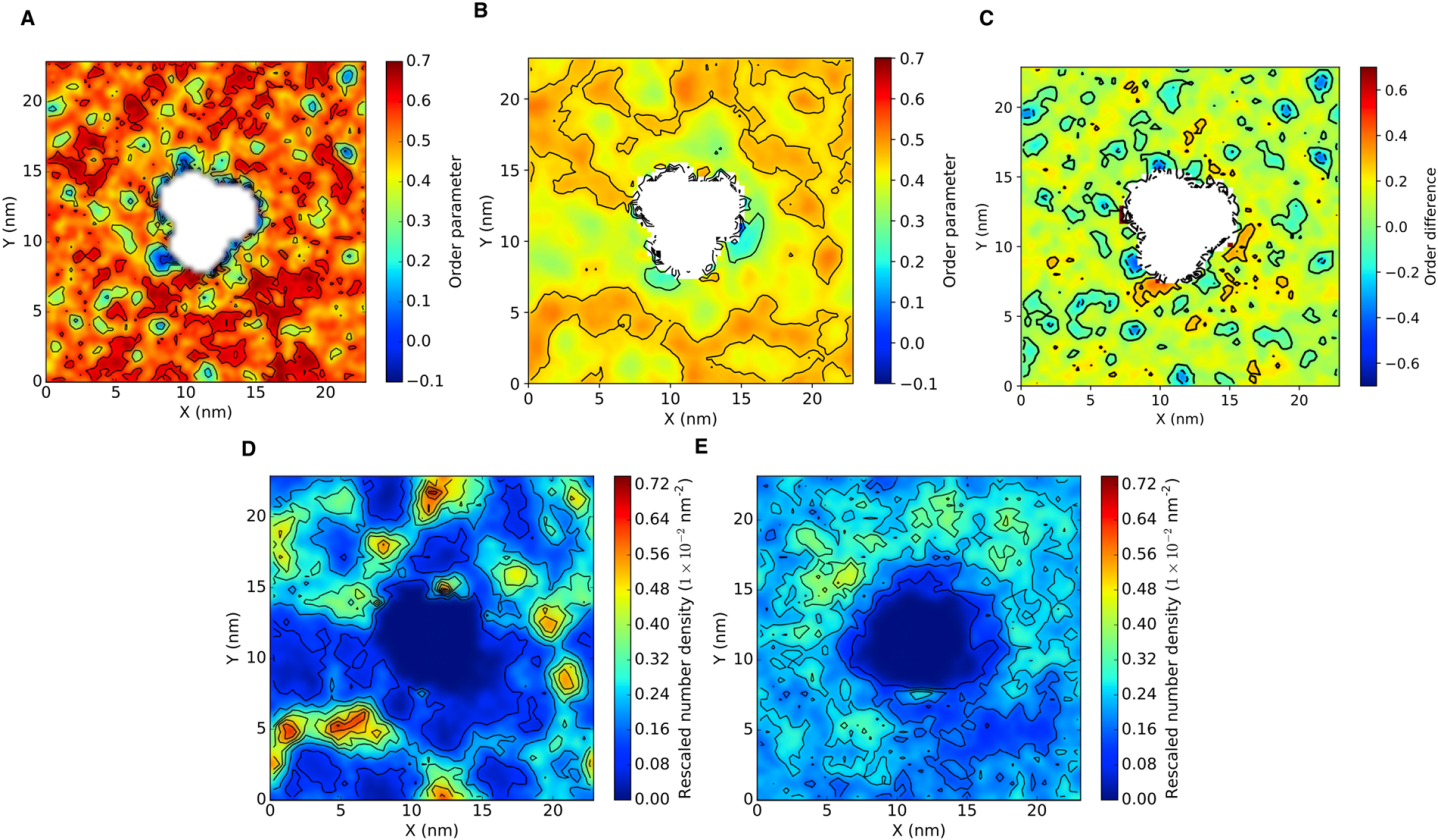
Order analysis of the lipid tails of (**A**) lipopolysaccharide, (**B**) POPE and the (**B**) difference between lipopolysaccharide and POPE order parameters in the outer membrane. (**C**,**D**) Density maps of cardiolipin in the outer and mixed phospholipid membrane, respectively. The density was measured using one phosphate particle per lipid and all values normalized by the number of lipids in a given leaflet. Each system was centered around a transmembrane OmpF.

Comparison to the outer membrane with an embedded OmpF shows quite similar trends (Fig. 4). We again observed clustering of cardiolipins below regions of high LPS disorder. Moreover, the regions of cardiolipin cluster corresponded to areas of high lower leaflet order, as observed for the OmpA system. The clustering of cardiolipin was more prevalent than the equivalent mixed phospholipid system, confirming that the observations were a result of the LPS layer. The degree of clustering was reduced compared to OmpA, which may suggest that LPS head group voids were smaller in the OmpF system. Thus while we do not observe any strong protein-lipid interactions in these systems, a protein may effect the packing of the LPS layer and thus the structure of the lower leaflet. We extended the OmpA simulation to 14 μs and while the trends in leaflet communication previously observed remained the same (SI, Figure 10), the number and size of cardiolipin voids increased and decreased, respectively. The trends in this section were also seen in the repeat simulations, as shown for the OmpA repeat in Figure S11.

## Discussion

In summary, we have shown the importance of membrane asymmetry towards the effect of a membrane protein on a lipid bilayer. The membrane thickness and order of lipid tails were reduced in a ~3 nm annulus around each protein for the symmetric mixed phospholipid membrane. Whereas for the asymmetric outer membrane the system properties are much more dependent on the structure and dynamics of the slow moving LPS layer. For the outer membrane an annulus of distinct membrane structure is not observed around each protein; instead LPS packing impacts the global structure of the membrane. Comparison between the structure and dynamics of outer membranes containing different protein and the pure lipid system suggest that the protein impacts the packing of the LPS layer and thus the properties of the lower leaflet. It is important to point out that while it is clear the proteins impact the packing of LPS, the significance of the nature of the protein is less clear. Thus for other membranes where the leaflet dynamics differ greatly the slowest layer will likely dominate the effects and importance of protein-lipid interactions. However, further work will be required to quantify this assertion.

## Methods

### Force field and model parameters

All simulations discussed here were performed using the Gromacs 5.1.4 MD package^27^, with the MARTINI 2.2 coarse-grained force field^20,21,28,29^.

For each outer membrane protein an X-ray structure was obtained from the Protein Data Bank (pdb codes are OmpA: 1QJP (0.165 nm resolution) and OmpF: 2OMF (0.240 nm resolution)) and any missing residues or broken loops were completed with Modeller^30^. The completed atomistic model was then coarse-grained with the Martinize script (Figure S1)^21^. OmpA is an 8 stranded beta barrel, which is very common in the outer membrane and is necessary for the structural integrity of the outer membrane^31,32^. The crystal structure has a length and diameter of approximately 5.6 and 2.2 nm, respectively. OmpF is a beta barrel trimer, which acts as an ion channel for positively charged ions^33^. In the crystal structure (pdb code 2OMF) each individual monomer had a length and diameter of approximately 5.1 and 3.3 nm and is composed of 16 beta strands.

### Simulation setup

Each membrane system (~23 × 23 × 15 nm) was generated with CHARMM-GUI^34–36^ and equilibrated until membrane properties converged (for up to 10 μs), using a 20 fs timestep. The outer membrane had an upper leaflet completely composed of lipopolysaccharide (LPS), while the lower leaflet was made up of 90% 16:0–18:1 phosphoethanolanime (POPE), 5% 16:0–18:1 phosphoglycerol (POPG) and 5% cardiolipin. The composition of the outer membrane was based on previous experimental and computational studies^16,37,38^. The symmetric mixed phospholipid membrane had the same composition as the inner leaflet of the outer membrane. LPS lipids were neutralized with Ca^2+^ ions; while any remaining charge imbalance was neutralized with Na^+^ and Cl^−^ ions. Each system contained ~2% antifreeze particles with respect to the number of water particles, to prevent any localized freezing of the solvent. Simulations were run at 313 K and the temperature maintained with a stochastic velocity rescale thermostat with a coupling constant of 1.0 ps. The pressure coupling was carried out using a semi-isotropic Parrinello-Rahman barostat, with a coupling constants of 12.0 ps.

The electrostatics were modeled with the reaction field method, using dielectric constants of 15 and infinity for the short range and long range regimes, respectively. The non-bonded forces were cutoff with the Potential-shift-verlet scheme. The short range cutoff for the electrostatics and non-bonded interactions was 1.2 nm. These simulations settings were based off a recent benchmark paper by associates of the  people responsible for the martini force field^39^.

After convergence of membrane properties each protein was inserted into the outer and mixed phospholipid membranes in a trans membrane orientation and any overlapping lipids were removed. An overlapping lipid was defined by whether it was within the concave hull of the protein or less than 0.1 nm from any protein bead. Each protein was inserted stochastically in a 2D disk in the xy plane of radius 3 nm, such that the overall change in lipid composition upon protein insertion was below 0.20%. For each system two 8 μs production runs were generated, using a 10 fs timestep. We extended all production runs by 6 μs in order to check for convergence of membrane properties after protein insertion. The analysis for these additional trajectories was included in the SI (Figures S10 and 12).

### Analysis of membrane properties

Structural analysis was carried out on the last 4 μs of each production run. The membrane 2D density, order and thickness maps were generated using tools developed by N. Castillo *et al*.^40^. The partial density map of a patch of a membrane was calculated using an in house script which utilized the MDAnalysis^41,42^ python module. The script was written to mimic the functionality of the gmx density gromacs tool, but could also analyze subsets of a system which do change size over time. The partial density analysis was carried out on the last 1 μs of each 8 μs production run.

## Electronic supplementary material

Supplementary information
