# Supplementary material for: Communication between the leaflets of asymmetric membranes revealed from coarse-grain molecular dynamics simulations
Source: Sci Rep. 2018 Jan 29;8:1805. doi: 10.1038/s41598-018-20227-1 (PMC5789016; doi:10.1038/s41598-018-20227-1)
Supplement: Supplementary file 1 — Supplementary information [file 41598_2018_20227_MOESM1_ESM.pdf]

Communication between the leaflets of asymmetric membranes revealed from coarse-grain molecular dynamics simulations.

Jonathan Shearer[1] and Syma Khalid[1*]

[1]University of Southampton, Southampton, SO17 1BJ, United Kingdom.

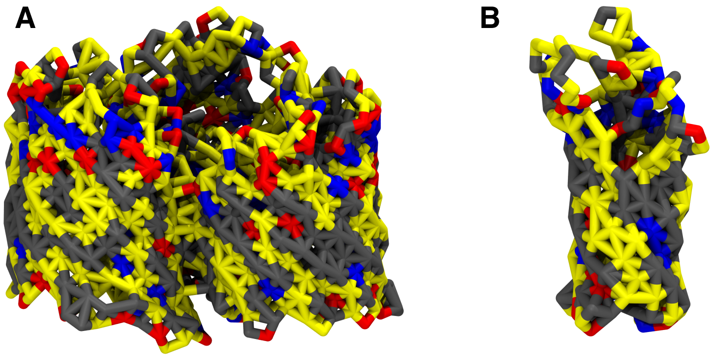

Figure S1. Diagrams of all of the proteins investigated in this study: A) OmpF and B) OmpA. Color key: grey = non-polar, yellow = polar, blue = positive charge and red = negative charge.

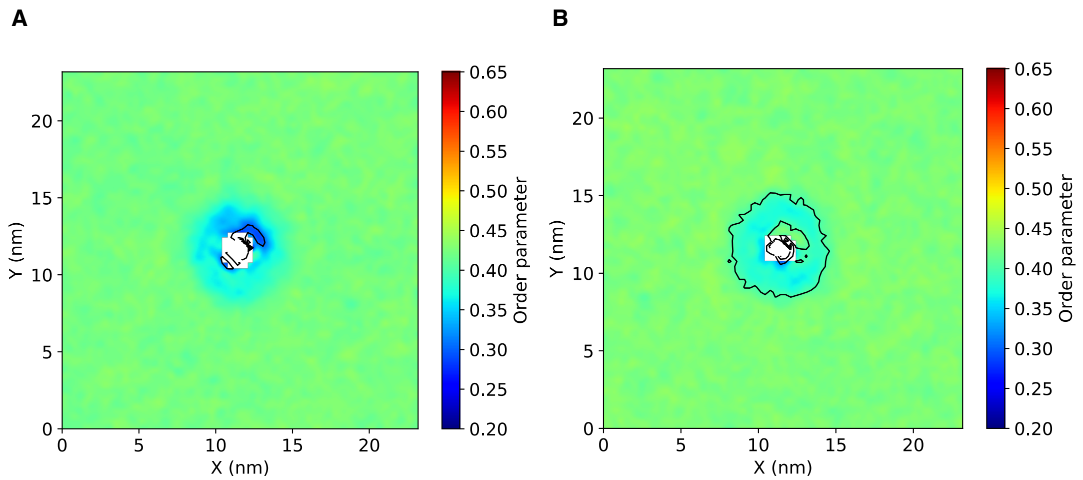

Figure S2. Order analysis map of the lipid tails of POPE in the A) upper and B) lower leaflet of a mixed phospholipid membrane centered on an embedded OmpA.

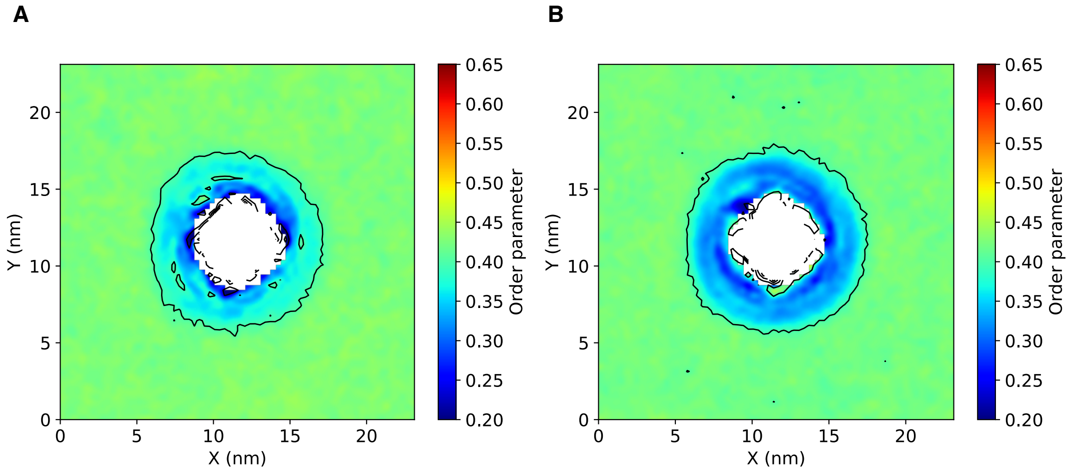

Figure S3. Order analysis map of the lipid tails of POPE in the A) upper and B) lower leaflet of a mixed phospholipid membrane centered on an embedded OmpF.

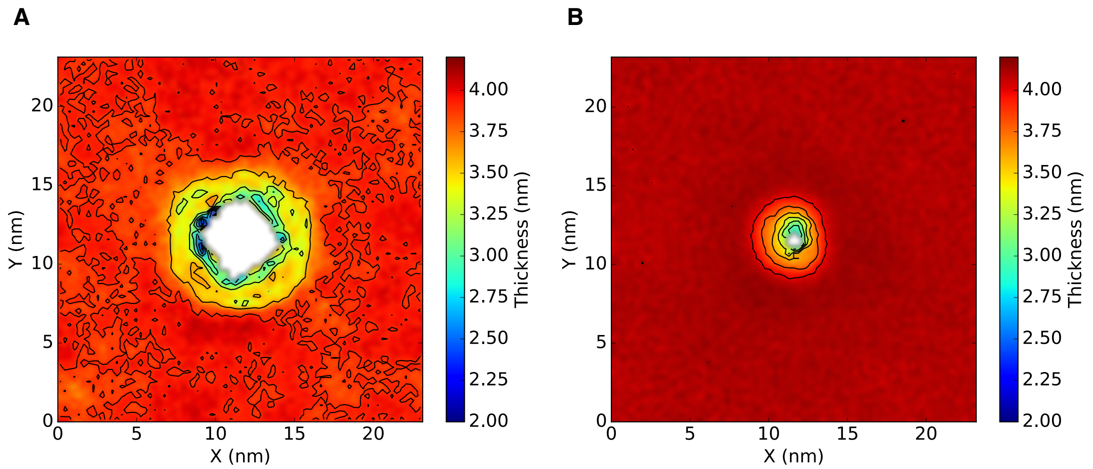

Figure S4. Membrane thickness map of a mixed phospholipid membrane centered on an embedded A) OmpF or B) OmpA. The thickness was measured using the phosphate beads (PO4, PO41 and PO42).

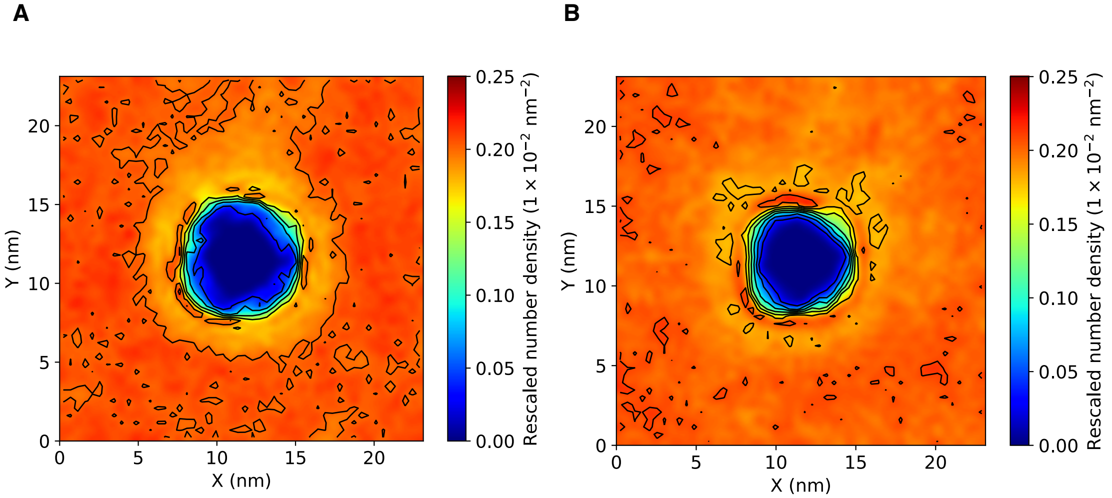

Figure S5. Density maps of POPE in A) upper and B) lower leaflets of the mixed phospholipid membrane centered around a membrane OmpF. The density was measured using the phosphate particle and all values normalized by the number of lipids in a given leaflet.

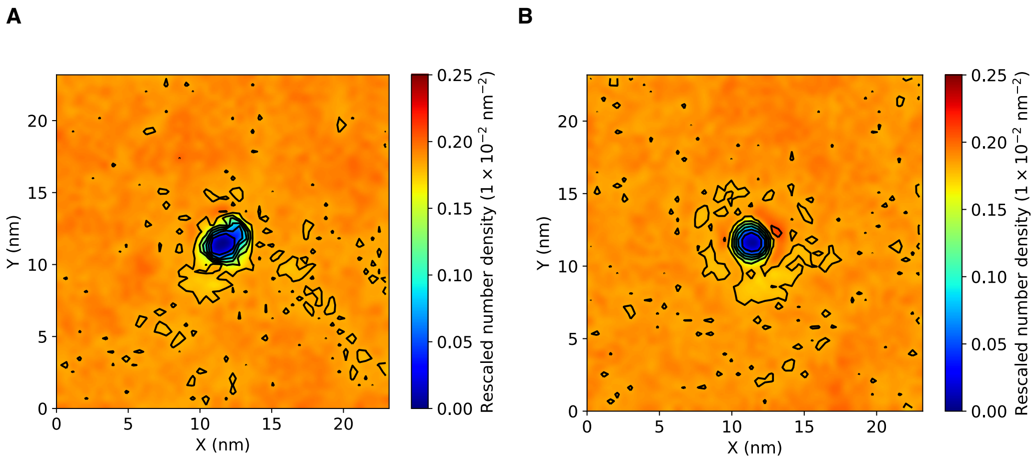

Figure S6. Density maps of POPE in A) upper and B) lower leaflets of the mixed phospholipid membrane centered around a membrane OmpA. The density was measured using the phosphate particle and all values normalized by the number of lipids in a given leaflet.

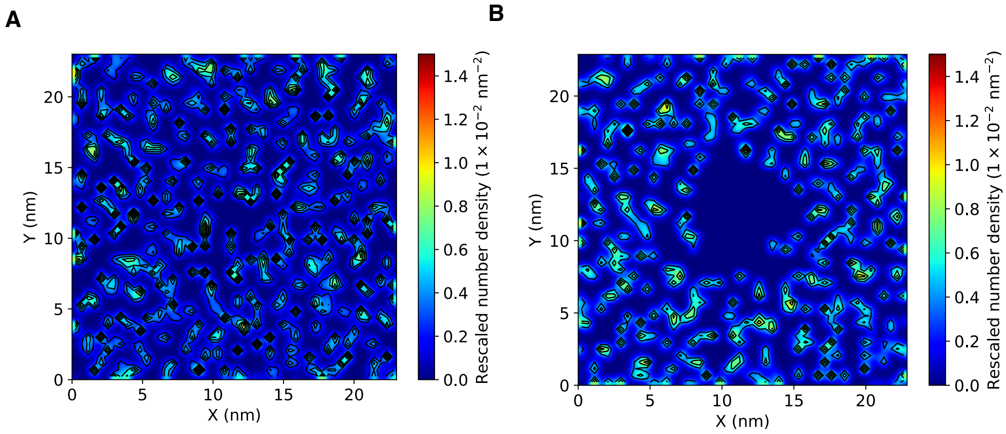

Figure S7. Density maps of LPS in the outer membrane systems containing A) OmpA and B) OmpF. The density was measured using a phosphate particle (PO1) per lipid and all values normalized by the number of lipids in a given leaflet. Trajectories were centered on the membrane protein.

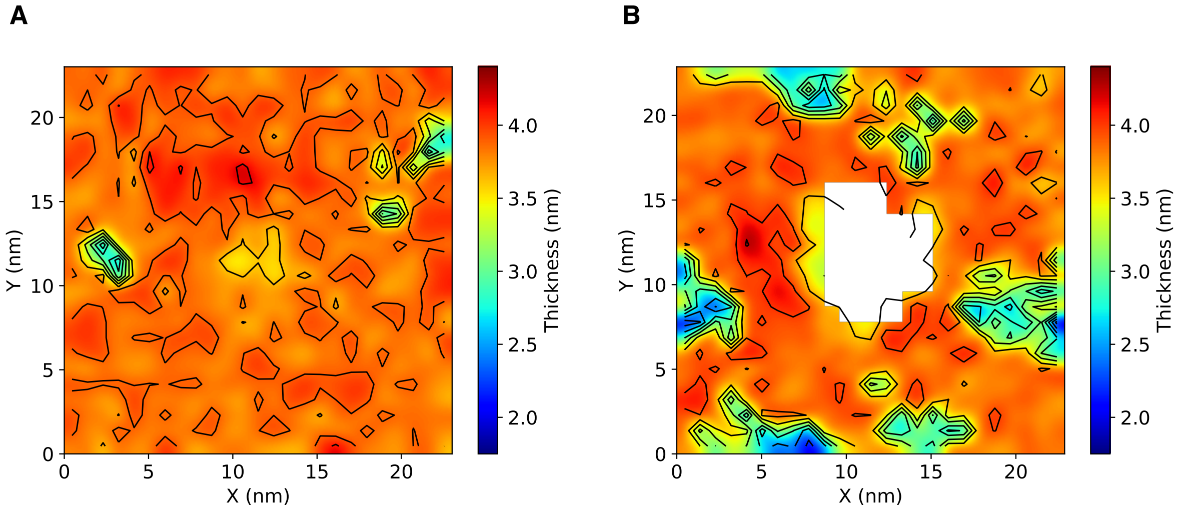

Figure S8. Membrane thickness map of the outer membrane centered on an embedded A) OmpA or B) OmpF. The thickness was measured using the phosphate beads (PO1, PO2, PO4, PO41 and PO42).

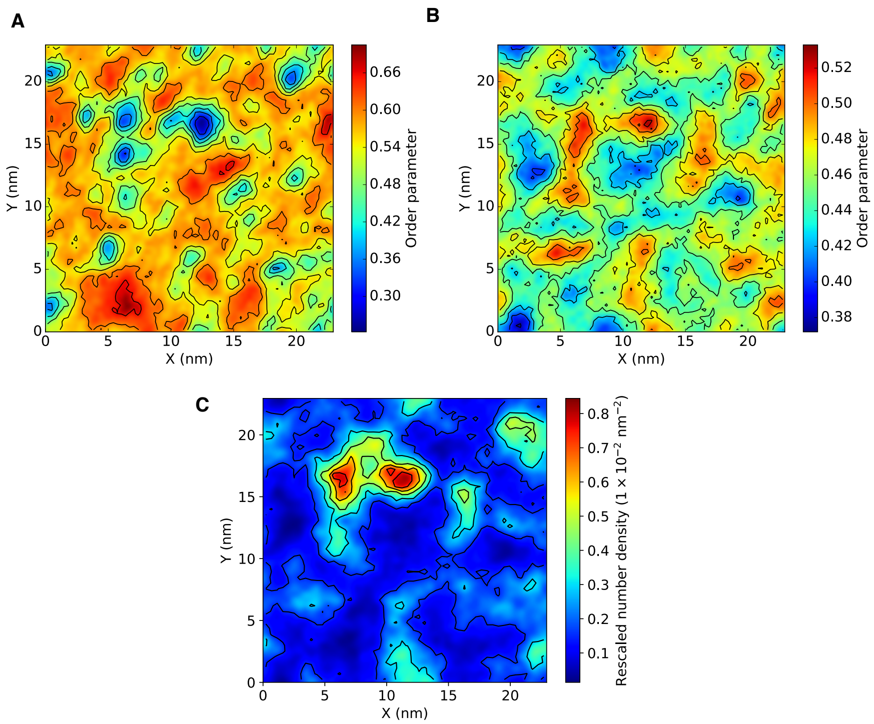

Figure S9. Order analysis map of lipid tails of A) LPS and B) POPE in the outer membrane. C) Density maps of cardiolipin in the outer and mixed phospholipid membrane, respectively. The density was measured using one phosphate particle per lipid and all values normalized by the number of lipids in a given leaflet.

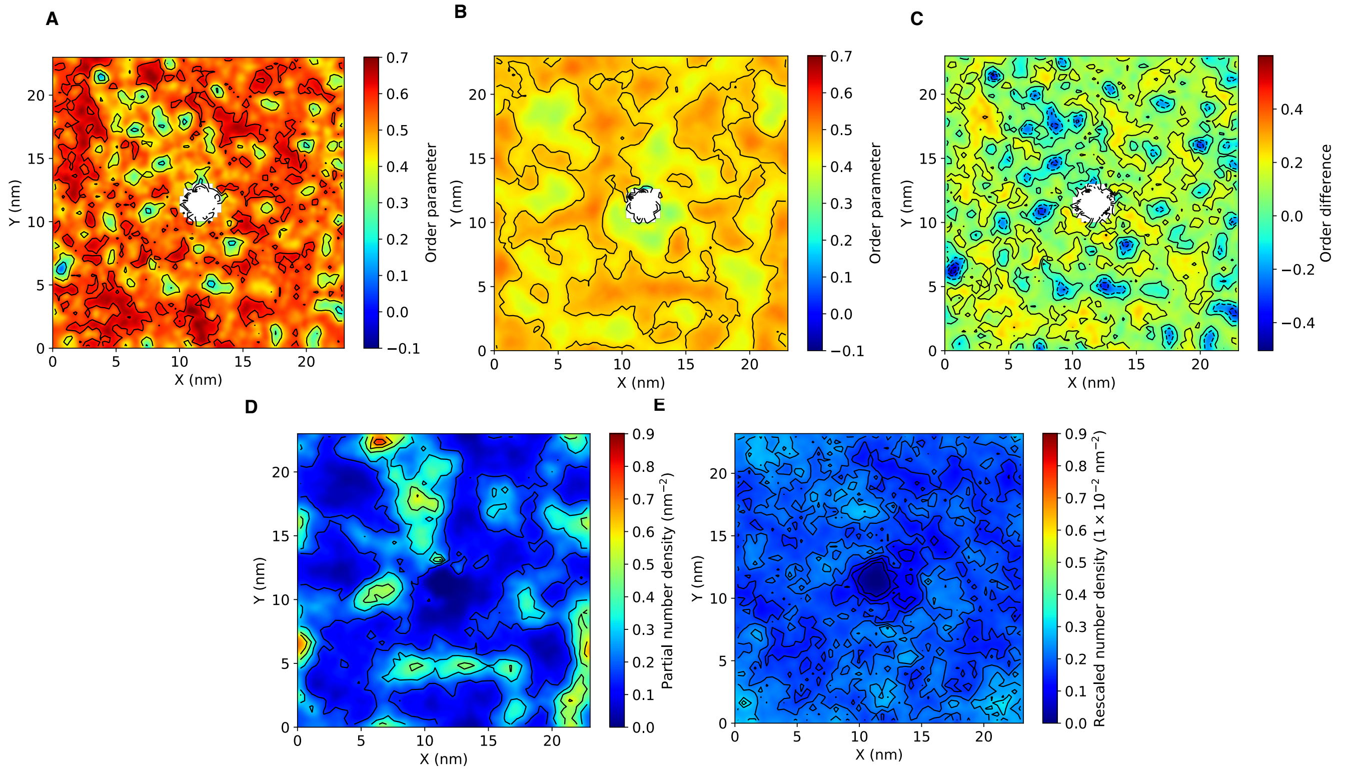

Figure S10. Order analysis of the lipid tails of A) lipopolysaccharide, POPE and the C) difference between lipopolysaccharide and POPE order parameters in the outer membrane. (D, E) Density maps of cardiolipin in the outer and mixed phospholipid membrane, respectively. The density was measured using one phosphate particle per lipid and all values normalized by the number of lipids. Each system was centered around a transmembrane OmpA. This is the analysis from the last 4 μs of an extended 14 μs simulation.

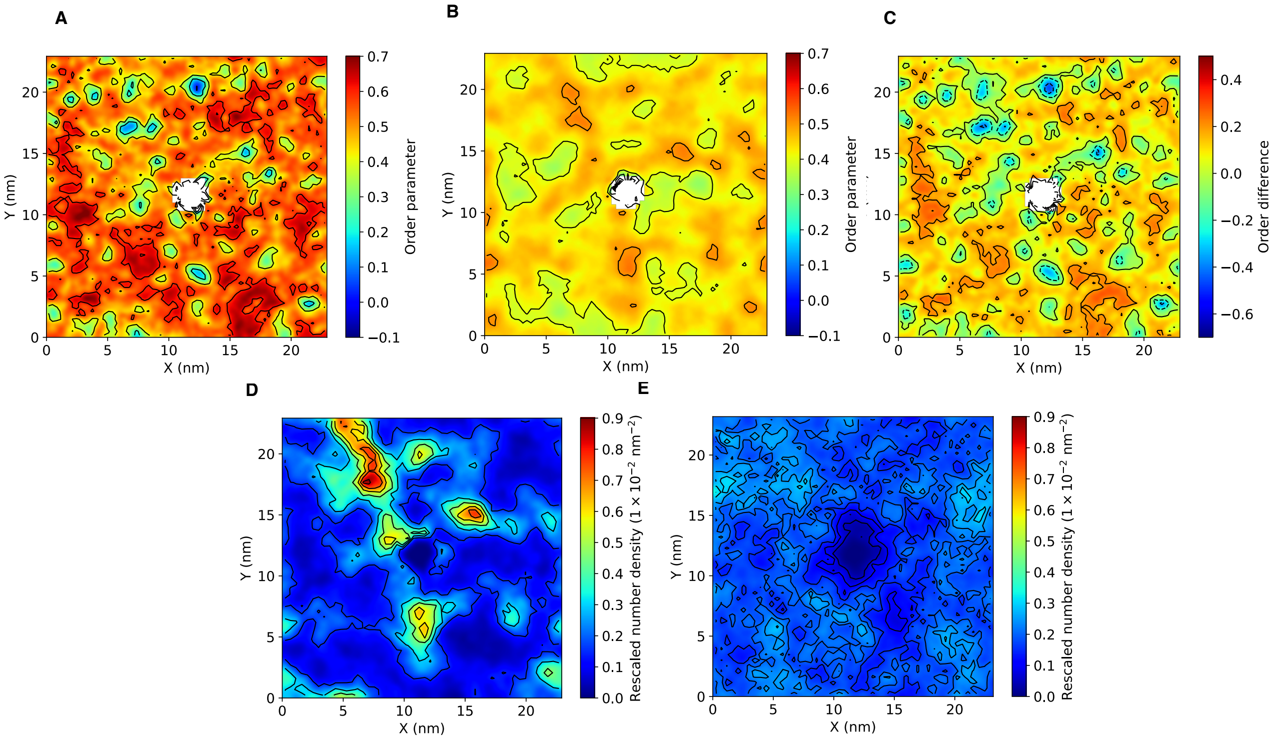

Figure S11. Order analysis of the lipid tails of A) lipopolysaccharide, POPE and the C) difference between lipopolysaccharide and POPE order parameters in the outer membrane. (D, E) Density maps of cardiolipin in the outer and mixed phospholipid membrane, respectively. The density was measured using one phosphate particle per lipid and all values normalized by the number of lipids. Each system was centered around a transmembrane OmpA. This is the analysis from the last 4 μs of the repeat simulation (8 μs total).

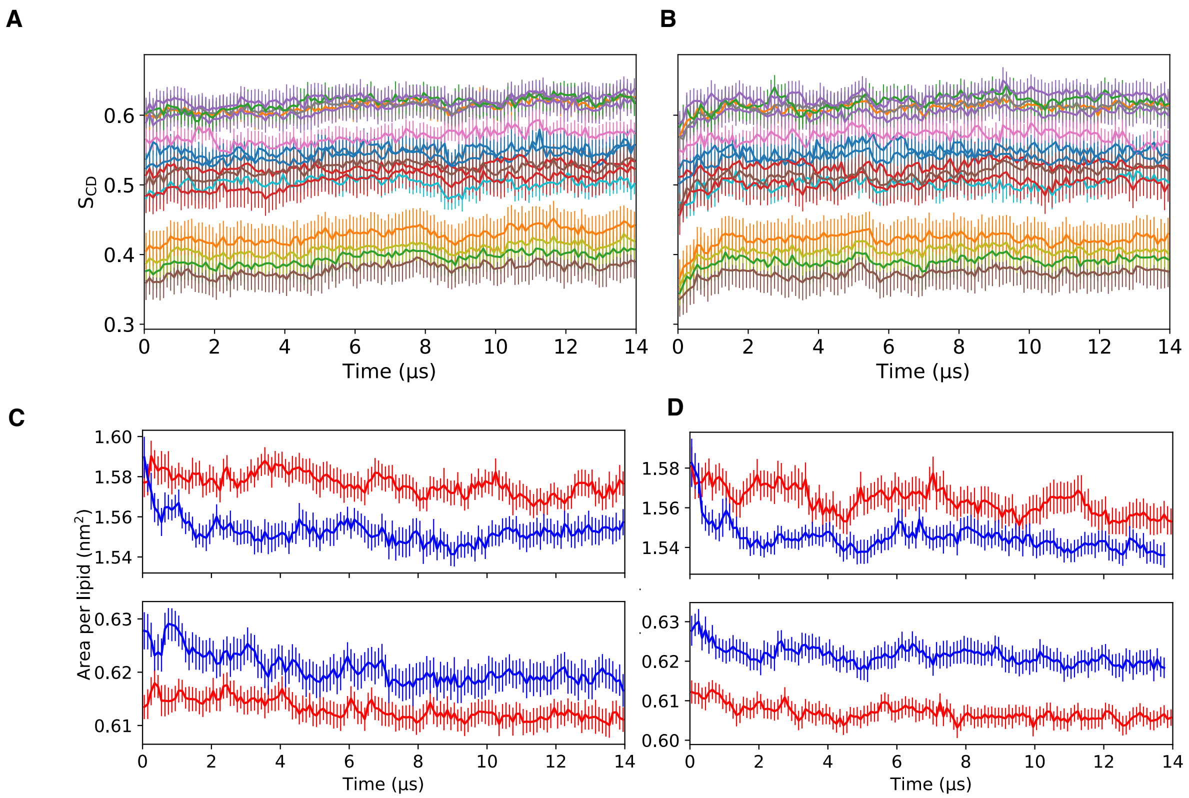

Figure S12. Deuterium order parameters convergence with respect to time for the outer membrane systems which contained A) OmpF and B) OmpA. C) The progression of the area per lipid for the LPS containing upper leaflet (top) and POPE in the lower leaflet (bottom) for an outer membrane with a membrane OmpF (blue) and OmpA (red). The area per lipid analysis was carried out for the repeat simulations. D) Progression of area per lipid for repeat simulations. Note that the plots and errors above were generated from the block average of 100 ns windows of each trajectory.
